# Novel immunotherapeutic approaches in gastric cancer

**DOI:** 10.1093/pcmedi/pbae020

**Published:** 2024-09-19

**Authors:** Meng Yang, Wuhao Lin, Jiaqian Huang, Alessandro Mannucci, Huiyan Luo

**Affiliations:** Department of Medical Oncology, State Key Laboratory of Oncology in South China, Collaborative Innovation Center for Cancer Medicine, Guangdong Provincial Clinical Research Center for Cancer, Sun Yat-sen University Cancer Center, Guangzhou 510060, China; Research Unit of Precision Diagnosis and Treatment for Gastrointestinal Cancer, Chinese Academy of Medical Sciences, Guangzhou 510060, China; Department of Molecular Diagnostics, State Key Laboratory of Oncology in South China, Collaborative Innovation Center for Cancer Medicine, Guangdong Provincial Clinical Research Center for Cancer, Sun Yat-sen University Cancer Center, Guangzhou 510060, China; Department of Medical Oncology, State Key Laboratory of Oncology in South China, Collaborative Innovation Center for Cancer Medicine, Guangdong Provincial Clinical Research Center for Cancer, Sun Yat-sen University Cancer Center, Guangzhou 510060, China; Research Unit of Precision Diagnosis and Treatment for Gastrointestinal Cancer, Chinese Academy of Medical Sciences, Guangzhou 510060, China; Gastroenterology and Gastrointestinal Emndoscopy Unit, IRCCS San Raffaele Hospital, Vita-Salute San Raffaele University, Milan 20132, Italy; Department of Molecular Diagnostics and Experimental Therapeutics, Beckman Research Institute of City of Hope; Monrovia, CA 91016, USA; Department of Medical Oncology, State Key Laboratory of Oncology in South China, Collaborative Innovation Center for Cancer Medicine, Guangdong Provincial Clinical Research Center for Cancer, Sun Yat-sen University Cancer Center, Guangzhou 510060, China; Research Unit of Precision Diagnosis and Treatment for Gastrointestinal Cancer, Chinese Academy of Medical Sciences, Guangzhou 510060, China

**Keywords:** gastric cancer, immunotherapy, immune checkpoint inhibitor, adoptive cell therapy, CAR-T

## Abstract

Gastric cancer is a malignant tumor that ranks third in cancer-related deaths worldwide. Early-stage gastric cancer can often be effectively managed through surgical resection. However, the majority of cases are diagnosed in advanced stages, where outcomes with conventional radiotherapy and chemotherapy remain unsatisfactory. Immunotherapy offers a novel approach to treating molecularly heterogeneous gastric cancer by modifying the immunosuppressive tumor microenvironment. Immune checkpoint inhibitors and adoptive cell therapy are regarded as promising modalities in cancer immunotherapy. Food and Drug Administration-approved programmed death-receptor inhibitors, such as pembrolizumab, in combination with chemotherapy, have significantly extended overall survival in gastric cancer patients and is recommended as a first-line treatment. Despite challenges in solid tumor applications, adoptive cell therapy has demonstrated efficacy against various targets in gastric cancer treatment. Among these approaches, chimeric antigen receptor-T cell therapy research is the most widely explored and chimeric antigen receptor-T cell therapy targeting claudin18.2 has shown acceptable safety and robust anti-tumor capabilities. However, these advancements primarily remain in preclinical stages and further investigation should be made to promote their clinical application. This review summarizes the latest research on immune checkpoint inhibitors and adoptive cell therapy and their limitations, as well as the role of nanoparticles in enhancing immunotherapy.

## Introduction

Gastric cancer (GC) casts a long shadow over global health, claiming >650 000 lives annually while affecting nearly one million patients. It is the fifth most common cancer globally and the fifth leading cause of cancer-related deaths [1]. For early GC, endoscopic resection is generally the preferred treatment option, often leading to favorable outcomes. However, because GC is rarely symptomatic in its early stages, by the time most patients experience symptoms, GC has progressed to an advanced stage. The prognosis for these patients is often grim, with an overall survival time of <6 months [2]. As a result, patients are often diagnosed with unresectable or metastatic GC, for which conventional systemic therapy has generally remained the only option to prolong survival. However, recent years have witnessed the birth of immunotherapy for GC, which marks a revolutionary turning point for the disease. This novel approach offers the promise of personalized precision medicine, bringing renewed hope to the fight against metastatic GC.

GC exhibits high molecular and phenotypic heterogeneity [3], which translates into four distinct molecular subtypes of GC, namely Epstein–Barr virus (EBV)-positive, microsatellite instability-high (MSI-H), genomically stable, and chromosomal instability [4]. About 10% of GCs are EBV-positive and these feature an abundant T-cell infiltration within the tumor microenvironment (TME) [5, 6]. Studies have demonstrated that programmed death-ligand 1 (PD-L1) inhibitors achieve an objective response rate (ORR) of >25% in this patient population [7, 8]. From an immunological perspective, lymphocyte-activation gene-3 (LAG-3) inhibitors offer promise in EBV-positive tumors due to their higher expression compared to EBV-negative GC in immunotherapy. These inhibitors on one hand expand and activate cytotoxic CD8^+^ T cells and, on the other hand, prevent the exhaustion of CD8^+^ T cells. These actions collectively enhance host immunity against the EBV virus. The incidence of MSI-H GC ranges from 6.6%–9.7% [9], typically correlating with advanced age, lower malignancy potential, and early tumor staging, and the mismatch-repair deficient (dMMR) is <5% in advanced GC [10]. Defects in mismatch repair (MMR) genes lead to the inability to repair DNA replication errors, ultimately causing MSI. Epigenetic silencing of human mutL homolog 1 (hMLH1) through promoter hypermethylation is the primary mechanism underlying dMMR deficiency [11]. This hypermutability translates to an increased number of mutations within the tumor cells, which in turn contributes to T-cell enrichment within the TME [12]. Tumors exhibiting MSI-H or dMMR status demonstrate favorable responsiveness to immunotherapy interventions. The CHECKMATE-649 immunotherapy trial, investigated the treatment outcome of advanced gastric cancer, gastroesophageal junction cancer, and esophageal adenocarcinoma with nivolumab [an anti-programmed death-receptor 1 (PD-1) monoclonal antibody] plus chemotherapy or chemotherapy alone. The subgroup of patients with MSI-H had a longer median overall survival (38.7 months) compared with those with microsatellite stable (MSS) GC (13.8 months) [13]. Likewise, patients with MSI-H GC receiving pembrolizumab-based therapy achieved an ORR > 40% [14]. Interestingly, the presence of high-density CD8^+^ and FOXP3^+^ tumor-infiltrating lymphocytes (TILs) can be considered potential prognostic markers for GC, as they are associated with a favorable prognosis [15]. Notably, chromosomal instability is another prevalent characteristic, which interacts with the TME and promotes heterogeneity in many cancer types, complicating treatment efforts and enabling cancer cells to evade therapies [16]. Chromosomal instability-type GC often modulates a complex tumor immune-suppressive microenvironment [17], and expresses genes involved in the PI3K/AKT signaling pathway and cell cycle process [18]. Some GCs develop tertiary lymphoid structures [19], which can shape the TME's inflammatory response, potentially serving as a future immunotherapeutic option. These molecular subtypes and their unique genetic features lay the foundation for patient-centered precision immunotherapy. Besides, TME and the associated immune cell response are influenced by various factors, including diet and the gut microbiome, which provides perspectives for synergistic or antagonistic study of immunotherapy [20, 21].

Motivated by recent breakthroughs in immunotherapy trials for metastatic GC and amidst a wave of advancements in metastatic GC treatment, this review mainly discusses two common types of immunotherapy. The first involves blocking inhibitory immune checkpoints in the TME to enhance the immune response of the body's own immune system, exemplified by immune checkpoint inhibitors (ICIs). The second approach involves culturing and modifying relevant immune cells *ex vivo* to activate anti-tumor effects, which are then transferred into the patient's body to exert their effects, such as adoptive cell transfer therapy. We will discuss the latest research on GC immunotherapy, with emphasis on the progress of adoptive cell transfer therapy.

## TME immune cells

**Figure 1.**
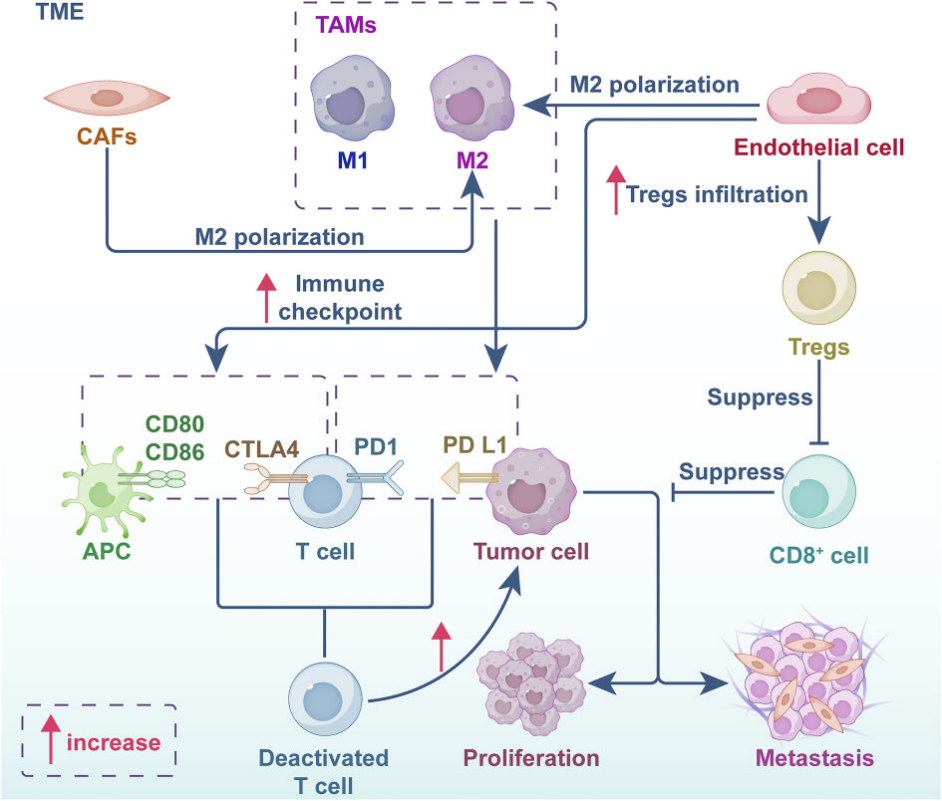
Cell interaction within the TME. Abbreviation: CAFs, cancer-associated fibroblasts; CTLA4, cytotoxic T-lymphocyte associated protein 4; M1, M1 macrophages; M2, M2 macrophages; PD-L1, programmed death-ligand 1; PD1, programmed death-receptor 1; TAMs, tumor-associated macrophages; TME, tumor microenvironment; Tregs, T regulatory cells.

The TME encompasses the internal and external environment surrounding tumor cells. It consists of various cell types, including tumor-associated fibroblasts (CAFs), immune cells [such as tumor-associated macrophages (TAMs) and myeloid-derived suppressor cells (MDSCs)], tumor-associated endothelial cells, and the extracellular matrix [22] (Fig. 1). The TME plays a critical role in immunotherapy efficacy by offering multiple targets for regulating tumor growth.

CAFs promote tumor development, and release cytokines, exosomes, and metabolites in a paracrine manner [23], ultimately leading to tumor chemotherapeutic resistance [24]. CAFs are recognized to exist in two subtypes: inflammatory CAFs (iCAFs) and extracellular matrix CAFs (eCAFs). These subtypes influence surrounding T cells and M2 macrophages, shaping a microenvironment conducive to tumor growth [25]. Furthermore, CAFs with high expression of SDC2 [26], INHBA-FAP [27], or other genes are associated with aggressive GC, poor prognosis, and distant metastasis. Notably, the GC risk factor *Helicobacter pylori* promotes progression by upregulating PIEZO1, which in turn increases CAF infiltration [28]. CAFs can also establish cross-talk with M2 macrophages. For example, CAFs secrete IGFBP7, which promotes the polarization of TAMs towards the M2 phenotype through the FGF2/FGFR1/PI3K/AKT signaling axis [29]. In fact, M2 macrophages are often associated with PD-L1 expression and contribute to immune escape and tumor growth [30, 31], while M1 macrophages promote anti-tumor T helper cell 1-mediated immune responses by expressing interleukin (IL)12 [32] and IL23 [33]. M2-like macrophages can further promote GC cell proliferation, metastasis, and even the transformation of normal cells into cancer cells through various signaling pathways, including the TNF-α/NF-κB/HIF-1α/miR-210/NTN4 axis [34] and the TGFβ2/NF-κB/Kindlin-2 axis [35]. High levels of CX3CR1 expression on MDSCs and TAMs can induce acute inflammatory responses and promote tumor progression [36].

TILs are a heterogeneous population primarily composed of T cells, B cells, and natural killer (NK) cells [37]. A specific subset of TILs, CD8^−^/4^−^ double-negative (DN) TILs, exhibits broad anti-tumor cytotoxic activity [38]. These DN TILs demonstrate superior tumor-homing ability while posing minimal off-target toxicity, making them promising candidates for adoptive cell transfer therapy in solid tumors [38]. Additionally, γδ T cells within the TME have also been shown to contribute to the suppression of GC progression [39].

T regulatory cells (Tregs), a subset of TILs, can suppress the immune response against cancer. They achieve this by inhibiting the proliferation, antigen presentation, and cytokine production of CD4^+^ and CD8^+^ T cells [40]. Tumor-infiltrating monocytic MDSCs (TI-M-MDSCs) are another population of immune cells within the TME with potent immunosuppressive capabilities [41]. These cells are particularly abundant in GC [42]. Notably, TI-M-MDSCs expressing the marker Immediate early response 3 (IER3) often exhibit stronger immunosuppressive abilities compared to their IER3-negative counterparts. The presence of IER3-positive TI-M-MDSCs is associated with a poorer prognosis for GC patients [42].

During GC development, endothelial cells express various vascular endothelial growth factor (VEGF) receptors, with ESM1 [43] and PDGFRB [44]. The expression of these molecules can modulate several cellular processes, including angiogenesis and leukocyte chemotaxis, therefore CAFs can modulate these events through signaling molecules like PGF, VEGFA, PDGF, and their corresponding receptors. Furthermore, endothelial cells with overexpression of THSD7A can promote GC cell proliferation and invasion, induce resistance to chemotherapy drugs, and contribute to an immunosuppressive microenvironment [45]. These effects are achieved through mechanisms including promotion of M2 macrophage and regulatory T cell infiltration, and upregulation of immune checkpoints like *HAVCR2, PDCD1LG2, TIGIT*, and*CTLA4*. In the context of *H. pylori* infection, bone marrow-derived mesenchymal stem cells can activate endothelial cells via the PI3K/AKT signaling pathway, leading to the formation of new tumor vessels within the TME [46]. Additionally, GC stromal stem cells may influence endothelial cells to express Slit2 [47], a positive regulator of tumor progression. In conclusion, the TME presents a multitude of potential targets for immunotherapy. However, further validation through experimental and clinical research is necessary.

## Immune checkpoint receptors

**Figure 2.**
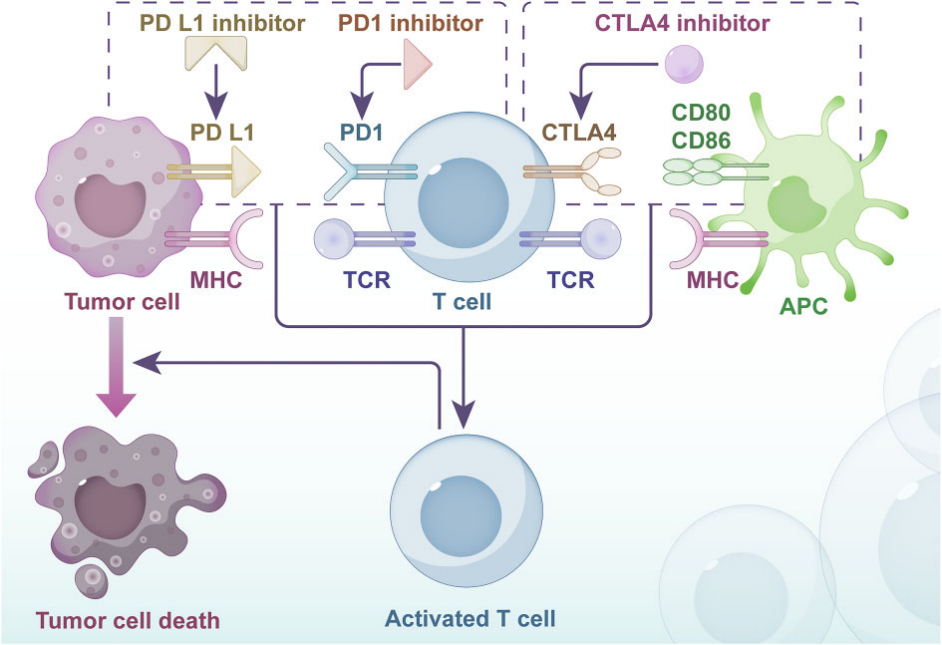
Mechanism of ICIs. ICIs block the small proteins such as PDL1 and CTLA4, produced by cancer cells and APC to activate T cells, which leads to tumor cell death. Abbreviations: APC, antigen presenting cell; CTLA4, cytotoxic T-lymphocyte associated protein 4; MHC, major histocompatibility complex; PD-L1, programmed death-ligand 1; PD1, programmed death-receptor 1; TCR, T cell receptor.

Immune checkpoint receptors, expressed on various immune cells, regulate immune system activation within a balanced range to prevent autoimmune diseases. These receptors interact with ligands [such as PD-L1, cytotoxic T-lymphocyte associated protein 4 (CTLA-4), LAG-3, T cell immunoreceptor with immunoglobulin and immunoreceptor tyrosine-based inhibition motifs (ITIM) domain (TIGIT)] on tumor cells or other immune-regulating cells, leading to inhibition of self-immunity and potentially enabling tumor immune escape. ICIs target these interactions to boost anti-tumor responses (Fig. 2). Nivolumab is a PD-L1 inhibitor that is combined with oxaliplatin for the treatment of metastatic GC. The ATTRACTION-4 trial demonstrated that this treatment combination could significantly improve progression-free survival compared to monotherapy in Asian patients with untreated, HER2-negative, unresectable advanced or recurrent gastric or gastroesophageal junction cancer (Hazard Ratio (HR) 0.68) [48]. In KEYNOTE-859, pembrolizumab plus chemotherapy significantly prolonged overall survival in patients with locally advanced or metastatic HER2-negative gastric or gastroesophageal junction adenocarcinoma compared to chemotherapy alone (12.9 months vs. 11.5 months; HR 0.78 [49], and has therefore met the requirements for first-line treatment recommendations [50]. Furthermore, the addition of ICIs to neoadjuvant chemotherapy improved the rates of histopathologic complete regression compared to neoadjuvant chemotherapy alone. Interestingly, these results were especially pronounced in the patients with PD-L1 Combined Positive Score (CPS) ≥ 10 and MSI subgroup [ 51]. CTLA-4 on T cells binds to antigen-presenting cell (APC) surface B7, keeping T cells in an inactivated state [52]. Dual-targeting ICIs, like cadonilimab, may target both PD-1 and CTLA-4. The COMPASSION-15 trial demonstrated that chemotherapy plus cadonilimab improved progression-free survival and overall survival in untreated HER2-negative patients with locally advanced or metastatic gastric or gastroesophageal junction adenocarcinoma compared to chemotherapy alone [53]. Additionally, MEDI5752, a bispecific antibody blocking CTLA-4 on PD-L1-positive cells [54], offers a novel approach with improved tumor targeting. LAG-3 represents another promising treatment target after PD-L1 and CTLA-4; it induces T cell depletion and blocks T cell proliferation, preventing anti-tumor responses [55]. A phase 1 dose-escalation study (DUET-4) is currently evaluating the safety and tolerability of the dual-specific antibody XmAb®22 841 (pavunalimab) targeting LAG-3/CTLA-4 in combination with pembrolizumab for specific late-stage solid tumors, but results have not been published (NCT03849469). Clinical trials related to LAG-3 are relatively few, and its therapeutic potential still needs further exploration. Overall, combination ICI therapy strategies appear more effective than single-agent therapy, and ICIs show promise in neoadjuvant and maintenance settings, highlighting the need for further research in these areas. Table 1 shows the Food and Drug Administration (FDA)-approved ICIs for the treatment of GC .

**Table 1.** FDA-approved ICIs for the treatment of GC and gastroesophageal junction cancer (GC/GEJ).

Drug (reference)	Target	Intervention/treatment	Cancer type	Initial approval year	Clinical trail
Nivolumab (Opdivo)	PD-1	Combination (nivolumab plus fluoropyrimidine- and platinum-containing chemotherapy)	Advanced or metastatic GC, GEJ, and esophageal adenocarcinoma	16 April 2021	CHECKMATE-649
Nivolumab (Opdivo)	PD-1	Alone	Completely resected esophageal or GEJ cancer with residual pathologic disease after neoadjuvant chemoradiotherapy	20 May 2021	CHECKMATE-577
Pembrolizumab (Keytruda)	PD-1	Combination (pembrolizumab plus trastuzumab, fluoropyrimidine, and platinum-containing chemotherapy)	Locally advanced unresectable or metastatic HER2-positive gastric or GEJ adenocarcinoma with PD-L1 expression (CPS ≥ 1)	16 May 2021	KEYNOTE-811
Pembrolizumab (Keytruda)	PD-1	Combination (pembrolizumab plus platinum and fluoropyrimidine-based chemotherapy)	Metastatic or locally advanced esophageal or GEJ carcinoma (tumors with epicenter 1 to 5 centimeters above the gastroesophageal junction)	22 March 2021	KEYNOTE-590
Pembrolizumab (Keytruda)	PD-1	Combination (pembrolizumab plus fluoropyrimidine- and platinum-containing chemotherapy)	Locally advanced unresectable or metastatic HER2-negative gastric or GEJ adenocarcinoma	16 November 2023	KEYNOTE-859

## Adoptive cell therapy

Adoptive cell therapy (ACT) is a personalized treatment option distinct from ICIs. Unlike ICIs that target inhibitory receptors on immune cells, ACT expands and modifies a patient's own immune cells *ex vivo* before reinfusing them to mediate anti-tumor functions. Through *ex vivo* manipulation, immune cells can acquire new functionalities by expressing novel receptors either endogenously or through gene modification, including the creation of chimeric antigen receptors (CARs) [56]. ACT has achieved significant clinical efficacy in treating hematologic malignancies. CAR-T cell therapy, for example, has shown the potential for long-term remission or complete remission in patients with blood cancers [57]. CAR-T targeting B-cell maturation antigen (BCMA) is FDA-approved for multiple myeloma, with ORR reaching 98% in phase I/II trials [58]. Similarly, CD19-targeted CAR-T therapy demonstrates efficacy in treating aggressive B-cell lymphomas [59] with ORR > 50%. However, ACT for solid tumors, including GC, remains in its infancy [60]. Several challenges hinder the therapeutic potential of ACT in GC [61]. These include tumor target antigen heterogeneity, difficulty in T cell infiltration, and the immunosuppressive nature of the TME [60, 61]. Over the past few decades, researchers have primarily focused on three forms of ACT: TILs, engineered T cell receptors (TCRs), and CAR T cells. Recently, there has been growing interest in modifying other immune effector cells using CAR engineering technology. This has led to the development of a new generation of ACT approaches, including CAR-NK cells, CAR-macrophages (MΦs), CAR-neutrophils, and CAR-natural killer T (NKT) cells. This section will explore the research progress of these diverse immune cell types in the context of ACT for GC treatment.

### CAR-T

**Figure 3.**
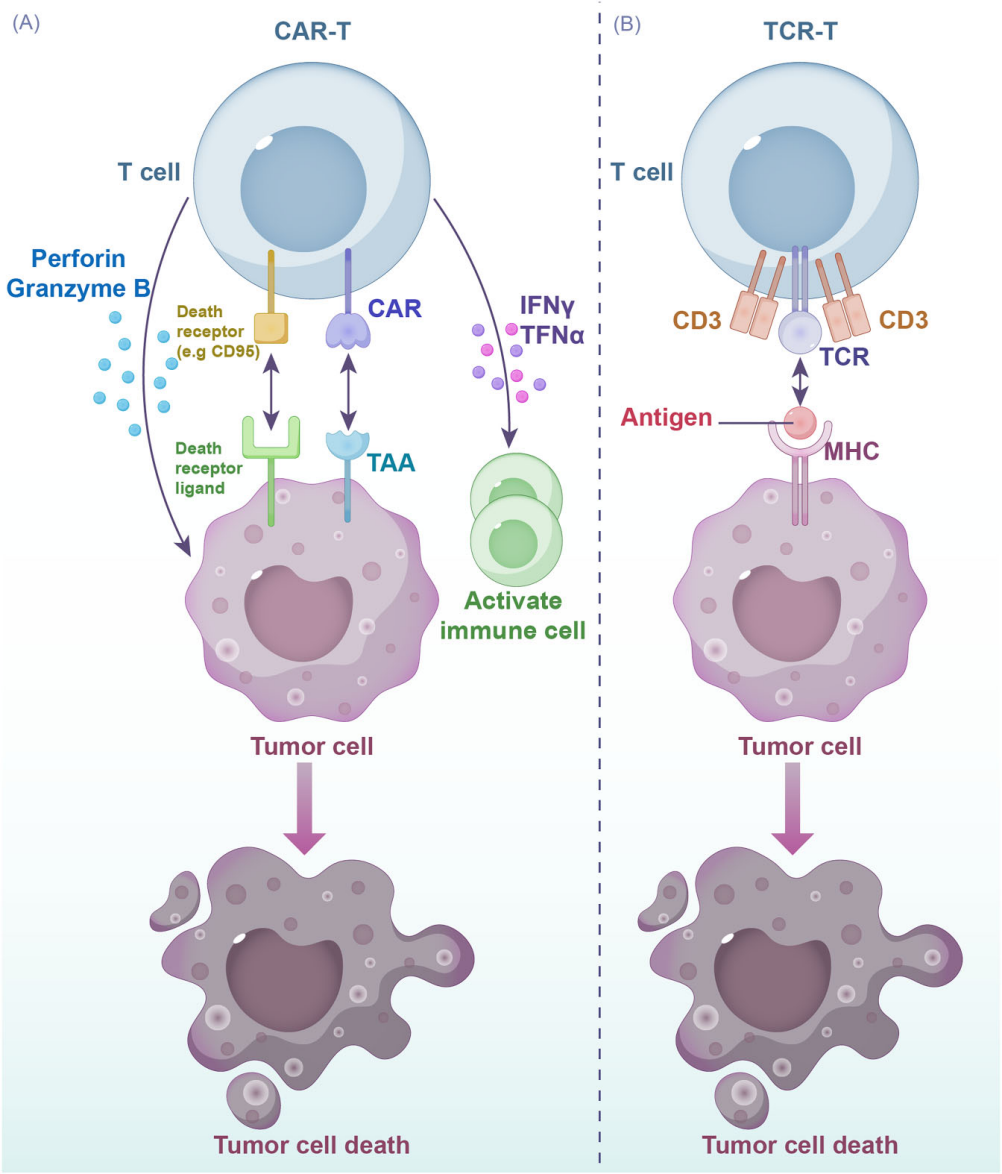
(**A**) Tumor cell eliminating mechanisms of CAR-T cells. CAR-T cells recognize tumor cells through the tumor associated antigen (TAA). Tumor cell death is mediated by activation of immune cell anti-tumor cytotoxic activity through interferon-gamma (IFN-γ) and tumor necrosis factor-alpha (TNFα) secretion, tumor cell apoptosis and necrosis activated through death receptor (e.g. CD95) recognition and perforin and granzyme B granules secretion. (**B**) Tumor cell eliminating mechanisms of TCR-T cells. TCR-T cells recognize tumor cell-derived antigens presented through MHC molecules and eliminate tumor cells through mechanisms discussed for (A).

CAR-T cells are engineered T cells designed to directly target specific antigens on the membrane surface of tumor cells. This bypasses the need for human leukocyte antigen (HLA) presentation, thereby enhancing the immune response (Fig. 3). CARs consist of four main parts: the antigen recognition domain, spacer region, transmembrane domain, and intracellular signaling domain [62]. The antigen recognition domain, expressed on the cell surface, is a single-chain variable fragment (ScFv) composed primarily of the variable light chain and heavy chain of immunoglobulins linked together by a serine-glycine linker [63]. CD28 and CD8a [64] are commonly used as the basic components of the spacer domain, providing flexibility for receptor–target binding. The transmembrane domain [65], instead has a hydrophobic α-helix structure and it promotes CAR expression and stability while activating downstream signaling proteins because it recruits and phosphorylates the intracellular signaling domain. αβ T cells are typically the preferred cell type for CAR-T therapy, achieving significant success in targeting CD19 B cell malignancies [66]. However, αβ T cell-based therapies may have significant limitations like cytokine-release syndrome (CRS), graft-versus-host disease (GVHD), poor infiltration of solid tumors, and immune escape, all of which require careful consideration. Conversely, γδ T cells share characteristics with NK cells, possessing both innate and acquired immune functions [67]. Unlike αβ T cells, γδ T cells recognize antigens independently of major histocompatibility complex (MHC) presentation, demonstrating superior tissue penetration and lower cytokine secretion [68]. CAR-γδT cells leverage these advantages to avoid the side effects and limitations typically associated with CAR-αβT cells [69]. In conclusion, both αβ and γδ T cells offer unique advantages and disadvantages. Personalized selection based on cancer type is crucial for optimal therapeutic outcomes.

The field of CAR-T cell therapy has advanced through four generations, each distinguished by the structure and composition of the intracellular domain. First-generation CAR-T cells [70], engineered with only the CD3ζ signaling domain, exhibited limited efficacy due to the lack of co-stimulatory signals necessary for T cell proliferation and anti-tumor function. The second generation addressed this limitation by incorporating co-stimulatory molecules like CD28 or CD137 [71] into the CAR design. Clinical trials using second-generation CAR-T cells, specifically those expressing anti-CD19 chimeric antigen receptors in autologous T cells, demonstrated robust T cell proliferation and significant efficacy against leukemia in patients with chronic lymphocytic leukemia. Further refinement through the inclusion of additional co-stimulatory domains, most commonly a combination of CD28 and 4–1BB [72], led to the development of the third generation. The fourth generation, also known as precision CAR or armored CAR [73], incorporates functionalities beyond just targeting tumor cells. These advanced CAR-Ts can be engineered to secrete immune-regulating factors, shaping the TME for a more potent anti-cancer response. The review will describe the common targets.

#### Her-2

When HER-2 is overexpressed on 10%–20% of GC cells [74], the GC often develops multidrug resistance [75, 76]. This observation has been a significant challenge for treatment for several years. In this context, the success of trastuzumab cannot be overstated as the first HER-2-targeted monoclonal antibody to successfully target HER-2-positive GC. However, this also highlights the potential of HER-2 as a target for CAR-T therapy [77]. Second-generation CAR-T cells utilizing lentiviral vectors to deliver a HER2-specific single-chain variable fragment along with CD3ζ and CD137 signaling domains have shown promise in preclinical studies [78]. These CAR-T cells actively suppress the tumorigenicity of GC stem-like cells in *in vitro* and *in vivo* xenograft models [78]. Notably, they exhibit long-term persistence and demonstrate directional migration towards tumor tissue [79]. Another preclinical study using subcutaneous xenograft and peritoneal metastasis models of GC investigated the therapeutic potential of HER2 CAR-T cells expressing a humanized chA21 ScFv and the co-stimulatory domain 4-1BBz [80]. *In vitro* studies confirmed that chA21-4-1BBz CAR-T cells significantly inhibited the growth of HER2-overexpressing tumors but had no effect on tumors with low HER2 expression [80]. Furthermore, these CAR-T cells demonstrated efficacy in targeting peritoneal cavity GC metastasis in xenograft mice, leading to tumor growth inhibition and prolonged survival [80]. Although most research on HER2 CAR-T therapy for GC remains in the preclinical stage, with limited evaluation in clinical trials, it holds promising prospects for future therapeutic development.

#### Claudin-18.2

Claudin-18.2 is a promising target for GC therapy due to its high and consistent overexpression in GC tissues [81]. Claudin-18.2 CAR-T cells, developed with claudin-18.2-specific ScFvs like hu8E5 and hu8E5-2I and incorporating CD28 co-stimulatory domains, have demonstrated efficacy in preclinical models [82]. These CAR-T cells effectively infiltrate and eliminate tumor tissues in xenograft models derived from both human and murine GC cell lines, with minimal damage to normal tissues [83]. Clinical trials support the promise of claudin-18.2 CAR-T therapy. A phase 1b study of CT041, a claudin-18.2 CAR-T product, reported the results of CT041 in a patient with metastatic GC who had failed multiple prior treatments. Following two CT041 infusions, the patient experienced a significant increase in CAR-T cell numbers, a decrease in circulating tumor DNA, and a partial response of gastric wall thickening and ascites, with no serious adverse effects [84]. This case suggests CT041’s potential to reduce the tumor burden to below minimal residual disease and achieve durable clinical responses. Interim results from another ongoing phase 1 clinical trial of CT041 in CLDN18.2-positive digestive system cancers are encouraging. This study reported an ORR of 48.6% and a disease control rate of 73.0%. Additionally, a significant proportion of patients maintained a sustained response at 6 months, with an ORR and disease control rate (DCR) of 57.1 and 75.0%, respectively. The overall survival rate at 6 months was also promising at 81.2% [85]. LY011, a third-generation anti-claudin18.2 CAR-T therapy, is currently under phase 1 investigation (NCT04966143 and NCT04977193). Early results from this study are encouraging, with an ORR of 50.0% and a DCR of 100% in patients with advanced solid tumors, including GC and pancreatic cancers. Importantly, no unexpected serious adverse reactions were observed [81]. In conclusion, claudin-18.2 CAR-T therapy demonstrates acceptable safety and promising anti-tumor efficacy in patients with GC.

#### Icam-1

Cell adhesion molecule 1 (ICAM-1) is often overexpressed in various malignancies, including GC [86]. This overexpression conveys a poorer prognosis and short survival (5-year disease-free survival rate: 36.1%, 5-year overall survival rate: 49.7% [86, 87]. Importantly, ICAM-1 expression is generally high across different molecular subtypes of GC, regardless of Lauren classification or TCGA subtyping [87]. *In vitro* studies using cell lines have demonstrated a positive correlation between the lytic activity of ICAM-1 CAR-T cells and the level of ICAM-1 expression on target cells. *In vivo* mouse models with GC receiving high-dose ICAM-1 CAR-T therapy showed a reduction in tumor burden, with some achieving complete eradication. However, most models also exhibited rapid relapse in 2 weeks [87]. To minimize off-tumor systemic toxicity, ICAM-1 CARs have been engineered to have a micromolar affinity (10 μM) [88]. These CARs specifically lyse cancer cells with high ICAM-1 expression while sparing normal tissues that express basal levels of ICAM-1. An open-label, multicenter trial investigated the safety and efficacy of a specific CAR-T therapy (IMC001) targeting epithelial cell adhesion molecule (EpCAM) as a surrogate antigen. Combining therapies targeting both EpCAM and ICAM-1 may offer amplified therapeutic effects [89]. However, challenges remain, including resistance development [90] and tumor relapse [87].

#### Carcinoembryonic antigen

Carcinoembryonic antigen (CEA) serves as a valuable tumor marker across various stages of cancer management, including screening, diagnosis, and prognosis [91]. Patients with GC often have elevated CEA levels as their disease progresses. Importantly, because of the ubiquitous nature of CEA, there has been a significant interest in the development of CEA-targeted CAR T cell therapy for advanced GC. Preclinical studies have demonstrated the safety and efficacy of CEA-CAR T cells, including their ability to inhibit tumor growth and extend lifespan [92]. A preclinical study exploring the potential of third-generation CAR structures incorporating humanized or fully human anti-CEA antibodies evaluated four different CEA-CAR designs for targeting CEA-positive cancers both *in vitro* and *in vivo* [93]. Furthermore, *in vitro* studies revealed a direct correlation between CEA expression in cancer cells and the cytotoxic activity of M5A, hMN-14, and BW431/26 CAR-T cells, with M5A demonstrating superior efficacy in mouse xenograft models [93]. Additionally, multiple studies have shown that combining CEA-CAR T cell therapy with cytokines, such as recombinant IL2 and IL12, can further enhance anti-tumor effects [94, 95]. Currently, seven clinical trials are investigating CEA-CAR T cell therapy for solid tumors and are actively recruiting participants (Table 2). A key focus of future research will be identifying the optimal ScFv with properties of stable expression and appropriate binding affinity.

**Table 2.** Summary of clinical trials of CAR T cell therapy in gastric cancer (available at https://clinicaltrials.gov, accessed on 1 June 2024).

Trail ID	Targeting antigen	Phase	Country	Estimated enrollment/actual enrollment	Selected population	Current status	Sponsor	Response	Toxicities (grade 3/4)
NCT06010862	CEA	1	China	36	Patients with CEA-positive advanced/metastatic solid tumors	Recruiting	Chongqing Precision Biotech Co., Ltd	-	-
NCT05415475	CEA	1	China	36	Patients with CEA-positive advanced malignant tumors	Recruiting	Chongqing Precision Biotech Co., Ltd	-	-
NCT05396300	CEA	1	China	60	Patients with CEA-positive advanced malignant solid tumors	Recruiting	Weijia Fang, MD	-	-
NCT04348643	CEA	1, 2	China	40	Patients with relapsed/refractory CEA + Cancer	Unknown	Chongqing Precision Biotech Co., Ltd	-	-
NCT02349724	CEA	1	China	75	Patients with CEA positive gastric cancer, lung cancer, pancreatic cancer, breast cancer and colorectal cancer	Unknown	Southwest Hospital, China	-	-
NCT06126406	CEA	1	China	60	Patients with CEA-positive advanced malignant tumors	Recruiting	Chongqing Precision Biotech Co., Ltd	-	-
NCT05538195	CEA	1, 2	China	60	Patients with CEA-positive advanced malignant tumors	Recruiting	Chongqing Precision Biotech Co., Ltd	-	-
NCT06006390	CEA	1, 2	China	60	Patients with CEA-positive advanced malignant tumors	Recruiting	Chongqing Precision Biotech Co., Ltd	-	-
NCT02416466	CEA	1	United States	6	Patients with CEA-expressing liver metastases	Completed	Roger Williams Medical Center	Overall Survival: 6.9 months (3.8–10.8+)	Colitis (2/6), fever (2/6), and edema (2/6)
NCT06043466	CEA	1	China	30	Patients with CEA positive advanced malignant solid tumors	Recruiting	Chongqing Precision Biotech Co., Ltd	-	-
NCT05620732	Claudin18.2	Not Applicable	China	20	Patients with advanced pancreatic cancer and gastric carcinoma	Recruiting	Shenzhen University General Hospital	-	-
NCT06134960	NKG2D/CLDN18.21	1	China	12	Patients with advanced NKG2DL+/CLDN18.2 + solid tumors	Not yet recruiting	Peking University	-	-
NCT05275062	Claudin18.2	1	China	6	Patients with advanced gastric/esophagogastric combination adenocarcinoma that has failed at least second-line therapy and advanced pancreatic cancer that has failed at least first-line therapy.	Unknown	Beijing Immunochina Medical Science & Technology Co., Ltd.	-	-
NCT05583201	NKG2D/CLDN18.21	1	China	18	Patients with advanced NKG2DL+/CLDN18.2 + solid tumors	Recruiting	Jianming Xu	-	-
NCT04581473	Claudin18.2	1, 2	China	192	Patients with advanced gastric/gastroesophageal junction adenocarcinoma and pancreatic cancer	Recruiting	CARsgen Therapeutics Co., Ltd.	-	-
NCT05539430	Claudin18.2	1	United States	56	Patients with unresectable, locally advanced, or metastatic gastric, gastroesophageal junction (GEJ), esophageal, or pancreatic adenocarcinoma	Recruiting	Legend Biotech USA Inc	-	-
NCT05472857	Claudin18.2	1	China	30	Patients with advanced solid tumors with positive CLDN18.2 expression	Recruiting	Suzhou Immunofoco Biotechnology Co., Ltd	-	-
NCT04404595	Claudin18.2	1, 2	United States	110	Patients with gastric, pancreatic cancer, or other specified digestive cancers	Active not recruiting	CARsgen Therapeutics Co., Ltd.	-	-
NCT06353152	Claudin18.2	1	China	12	Patients with gastric/gastroesophageal junction adenocarcinoma	Recruiting	Peking University	-	-
NCT05277987	Claudin18.2	1	China	18	Patients with advanced gastric/esophagogastric junction adenocarcinoma and pancreatic cancer	Recruiting	Shenzhen Fifth People's Hospital	-	-
NCT05393986	Claudin18.2	1	China	63	Patients with solid tumors	Recruiting	Peking University	-	-
NCT04977193	Claudin18.2	1	China	18	Patients with advanced gastric adenocarcinoma	Recruiting	Shanghai Longyao Biotechnology Inc., Ltd.	-	-
NCT05952375	Claudin18.2	Not Applicable	China	9	Patients with claudin 18.2 positive advanced gastric cancer	Recruiting	The Affiliated Hospital of Qingdao University	-	-
NCT03890198	Claudin18.2	1	China	2	Patients with advanced gastric cancer and pancreatic ductal adenocarcinoma	Terminated	First Affiliated Hospital Xi'an Jiaotong University	-	-
NCT03874897	Claudin18.2	1	China	37	Patients with previously treated, CLDN18.2-positive digestive system cancers	Completed	Peking University	Patient with gastric cancer: Objective Response Rate: 57.1%, Disease Control Rate: 75.0%, respectively, and the 6-month overall survival rate was 81.2%	Hematologic toxicity (37/37)
NCT03159819	Claudin18	Not Applicable	China	24	Patients with advanced gastric adenocarcinoma and pancreatic adenocarcinoma	Unknown	Changhai Hospital	-	-
NCT06055439	Cadherin 17	1, 2	United States	135	Patients with advanced gastrointestinal (GI) cancers that are relapsed or refractory to at least 1 standard treatment regimen in the metastatic or locally advanced setting.	Not yet recruiting	Chimeric Therapeutics	-	-
NCT04503278	Claudin 6	1	Australia	145	Patients with CLDN6-positive relapsed or refractory advanced solid tumors	Recruiting	BioNTech Cell & Gene Therapies GmbH	-	-
NCT02725125	EPCAM	Not Applicable	China	19	Patients with relapsed or refractory gastric cancer	Unknown	Sinobioway Cell Therapy Co., Ltd.	-	-
NCT03563326	EPCAM	1	China	40	Patients with advanced gastric cancer and peritoneal metastasis	Unknown	Jian-Kun Hu	-	-
NCT03013712	EpCAM	1, 2	China	60	Patients with EpCAM-positive cancer	Unknown	First Affiliated Hospital of Chengdu Medical College	-	-
NCT02915445	EpCAM	1	China	30	Patients with nasopharyngeal carcinoma, breast cancer and other EpCAM positive solid tumors.	Active not recruiting	Sichuan University	-	-
NCT05028933	EpCAM	1	China	48	Patients with malignant tumors of the digestive system	Recruiting	Zhejiang University	-	-
NCT02713984	HER2	1, 2	China	0	Parients with HER2-positive cancer	Withdrwan	Zhi Yang	-	-
NCT04650451	HER2	1	United States	220	Patients with HER2-positive solid tumors	Suspended	Bellicum Pharmaceuticals	-	-
NCT04511871	HER2	1	China	15	Patients with relapsed or refractory HER2-positive solid tumors	Recruiting	Shanghai PerHum Therapeutics Co., Ltd.	-	-
NCT03740256	HER2	1	United States	45	Patients with advanced HER2-positive solid tumors	Recruiting	Baylor College of Medicine	-	-
NCT03941626	Mesothelin	1, 2	China	50	Patients with solid malignancies	Unknown	Shenzhen BinDeBio Ltd.	-	-
NCT03638206	Mesothelin	1, 2	China	73	Patients with malignancy gastric cancer	Unknown	Shenzhen BinDeBio Ltd.	-	-
NCT04847466	PD-L1	2	United States	55	Patients with recurrent/metastatic gastric or head and neck cancer	Recruiting	National Cancer Institute (NCI)	-	-
NCT02862028	PD-L1	1, 2	China	20	Patients with EGFR family member-positive advanced solid tumor	Unknown	Shanghai International Medical Center	-	-
NCT02617134	MUC1	1, 2	China	20	Patients with MUC1-positive relapsed or refractory solid tumor	Unknown	PersonGen BioTherapeutics (Suzhou) Co., Ltd.	-	-
NCT05239143	Mucin1 cell surface associated C-Terminal (MUC1-C)	1	United States	100	Patients with advanced or metastatic solid tumors	Recruiting	Poseida Therapeutics, Inc.	-	-
NCT03960060	tyrosine kinase-like orphan receptor 2 (ROR2)	1	China	18	Patients with relapsed and refractory stage IV metastatic solid tumors	Unknown	Shanghai PerHum Therapeutics Co., Ltd.	-	-
NCT04864821	Cd276 (B7-H3)	1	Not provided	24	Patients with CD276-positive advanced solid tumor	Unknown	PersonGen BioTherapeutics (Suzhou) Co., Ltd.	-	-
NCT04107142	NKG2DL	1	Malaysia	10	Patients with relapsed or refractory solid tumour	Unknown	CytoMed Therapeutics Pte Ltd	-	-
NCT04427449	CD44v6	1,2	China	100	Patients with CD44v6-positive cancer	Unknown	Shenzhen Geno-Immune Medical Institute	-	-

#### c-Met

Cellular mesenchymal epithelial transition factor (c-Met), also known as hepatocyte growth factor receptor, is frequently overexpressed in various cancers [96]. This overexpression contributes to significantly reduced overall survival by promoting abnormal signaling pathways that support tumor cell development and metastasis [97]. Consequently, the c-Met signaling pathway has emerged as an attractive target for GC therapy. Preclinical studies have demonstrated the promise of c-Met-directed CAR-T cells. Two types of CAR-T designs incorporating an anti-c-Met scFv, CD28 costimulatory domain, and CD3ζ signaling domain (c-Met CAR KHYG-1 and c-Met CAR Jurkat) have shown antitumor effects in both *in vitro* and *in vivo* models, with c-Met CAR KHYG-1 cells exhibiting superior cytotoxic activity [98]. Furthermore, CAR-T cells co-expressing PD1/CD28 chimeric signaling receptor can improve the immunosuppressive TME of solid tumors [99], leading to enhanced antitumor efficacy. Dual-function chimeric antigen receptor T cells targeting both c-Met and PD1 have demonstrated stronger antitumor activity in PD-L1-positive tumor xenograft models compared to single-target CAR-T cells [100]. This dual CAR strategy holds promise for minimizing off-target toxicity to normal tissues, although further research is warranted.

#### L1 cell adhesion molecule

The L1 cell adhesion molecule (L1CAM), a protein highly expressed in various malignant tumors, is closely associated with tumor invasion, metastasis, and poor prognosis. L1CAM CAR-T cell therapy, also known as CX804 [101], utilizes a novel ScFv called H8 ScFv to target L1CAM. Preclinical studies have shown that CX804 can effectively eliminate patient-derived cells with high L1CAM expression. Furthermore, CX804 demonstrates cytotoxic activity against GC cell lines MKN-28 and adenocarcinoma gastric cell line (AGS). Additionally, CX804 has been shown to promote immune cell activation by secreting T cell activation factors such as interferon-gamma (IFN-γ) and tumor necrosis factor alfa (TNF-α) [101].

#### EpCAM

EpCAM is a protein highly expressed on the surface of various digestive system tumors. Decreased EpCAM expression can lead to reduced proliferation and cell cycle arrest in AGS cells, hindering GC formation [102]. Consequently, EpCAM has emerged as a promising target for clinical cancer therapy. Early-phase human trials for patients with late-stage colorectal and gastric cancer have demonstrated the safety and efficacy of EpCAM CAR-T cell therapy (specifically, IMC001) [103]. These trials observed stable engraftment, meaning the CAR-T cells persisted and functioned within the patients' bodies, along with increased levels of cytokines, which are immune signaling molecules.

#### Other targets

In addition to the previously mentioned targets, several other molecules are being explored for CAR-T therapy in GC. CD133CAR-T cells [104], when combined with cisplatin treatment, can eliminate CD133-positive BGC-823 cells, potentially improving treatment outcomes. Preclinical studies suggest that CDH17CAR-T cells are effective in inhibiting the growth of neuroendocrine and gastrointestinal tumors [105]. Similarly, CAR-T cells targeting GPC3 [106] show promising antitumor activity in xenograft models of aggressive solid tumors. FOLR1, a protein overexpressed in GC tissues but with low expression in normal tissues, is considered a potential target for immunotherapy. Studies have demonstrated that FOLR1-directed CAR-T cells can induce cytotoxic effects on FOLR1-positive GC cells [107]. Anti-mesothelin CAR-T cells [108], such as the M28z10 T cells [108], have been shown to induce tumor regression in various xenograft mouse models of GC. PD-L1 [109], mucin1 (MUC1) [110], cadherin 17 [111], B7H3 [112], TEM8 [113], and prostate stem cell antigen (PSCA) [114] are among other potential GC-related CAR-T targets currently under investigation. Table 2 summarizes the clinical trials of CAR T cell therapy in GC up until 1 June 2024.

### TCR-T

TCR-T cell therapy offers a promising approach for cancer treatment. Unlike CAR-T cells, which recognize tumor cell surface antigens directly, TCR-T cells exhibit a higher degree of specificity. They recognize cancer cell-derived antigens presented through the HLA complex, allowing for targeted elimination of tumor cells with minimal damage to healthy tissues [115]. This specificity makes TCR-T therapy potentially more suitable for solid tumor treatment. However, TCR-T cell therapy has limitations. The recognition of target antigens by TCR-T cells depends on their presentation by MHC molecules [116]. This restricts TCR-T efficacy to patients with specific HLA alleles that can effectively present the relevant epitopes (antigenic fragments). The HLA-A*02:01 allele is one of the most commonly studied HLA types in this context. TCR-T cells exhibit a higher level of sensitivity compared to CAR-T cells [117]. TCR-T cell activation can be triggered by stimulation from a single MHC–antigen complex, whereas CAR-T cells typically require engagement with thousands of epitopes to induce a robust immune response (Fig. 3). Structurally, TCRs consist of alpha and beta chains that form a disulfide-linked heterodimer [118]. This complex, along with CD3εγδζ hexamers [116], forms an octameric structure on the T cell surface. TCR binding to an MHC–antigen complex leads to the phosphorylation of immunoreceptor tyrosine-based activation motifs within the CD3 subunits [119]. This phosphorylation cascade ultimately leads to T cell activation and effector functions. TCR-T therapy can target a variety of tumor antigens, including tumor-associated antigens (TAAs), cancer-germline antigens (CGAs), and tumor-specific antigens (TSAs) [120]. This versatility offers the potential for broader applicability in cancer treatment. TAAs are antigens found on both tumor cells and healthy tissues, but because tumor cells express them to significantly higher levels, untoward side effects are not too common. These TAAs can be further categorized as differentiation antigens (shared with the tissue of origin) or overexpressed antigens. Clinical trials are currently underway to evaluate the safety and efficacy of TCR-T cells targeting the TAA KK-LC-1 [121] in metastatic gastric, lung, breast, and cervical cancers (ClinicalTrials.gov IDNCT05483491). CGAs are attractive targets due to their limited expression in normal tissues, making them, essentially, antigens whose expression is restricted to cancer cells and germline cells [122]. TSAs, finally, are often associated with gene mutations or viral infections, and they represent ideal targets due to their complete absence in healthy tissues, minimizing the risk of off-target toxicity [123]. However, identifying these neoantigens can be challenging due to patient-specific mutations and the unpredictable nature of MHC affinity. Despite the reduced off-tumor toxicity compared to CAR-T therapy, TCR-T therapy still faces challenges [124] such as selection of targeted antigen, limited TCR-T cell persistence, immunosuppressive TMEs, and tumor immune escape mechanisms. To address these shortcomings, preclinical evaluations are crucial. These evaluations assess HLA compatibility, TCR affinity, and safety profiles to optimize TCR-T therapy for individual patients.

### TILs

TILs residing within the TME are often rendered dysfunctional by the activation of immune checkpoint receptors [125] such as PD-L1, and CTLA-4, and signaling pathways like the WNT-β-catenin pathway [126]. These factors contribute to reduced T cell infiltration and activation, while promoting T cell exhaustion, ultimately leading to tumor immune evasion. TIL therapy offers a promising approach to overcome this challenge. It involves the extraction of lymphocytes directly from a patient's tumor and their expansion *ex vivo* in a culture enriched with IL-2, anti-CD3 monoclonal antibodies, and allogeneic feeder cells before their eventual re-infusion for treatment purposes. This process aims to reverse the suppressive state of these TILs and enhance their anti-tumor capabilities. Reinfused into the patient, these activated TILs can then mount a targeted attack against the tumor. TIL therapy has demonstrated success in treating melanoma, with lifileucel [127], a TIL therapy for advanced melanoma following PD-1/PD-L1 therapy, receiving accelerated FDA approval. Early-phase clinical trials targeting GC have also shown promise [128]. However, significant breakthroughs for GC treatment are still needed. A key focus for improving TIL therapy in GC is the identification of tumor neoantigens. These are antigens unique to the patient's tumor and highly immunogenic, meaning they can trigger a strong immune response. Identifying neoantigens with high antigen load can significantly enhance TIL sensitivity and anti-tumor efficacy within the TME. Combination therapy strategies incorporating ICIs [129], like PD-L1 blockade or genetic knockout of PD-L1 using CRISPR/Cas9 technology [130], have also shown promising clinical results in non-melanoma tumors. TIL therapy offers several advantages. It has minimal immune-mediated and off-target toxicities. However, challenges remain. The high cost, complex manufacturing process, potential adverse events associated with lymphocyte depletion and systemic IL-2 administration prior to TIL infusion, and the risk of autoimmune reactions post-infusion require further optimization for broader clinical application.

### CAR-NKs

NK cells act as critical regulators of cellular activity through a balance of inhibitory and activating surface receptors. Healthy cells express MHC I molecules [131] which are recognized by inhibitory NK cell receptors. This recognition mechanism prevents NK cells from attacking healthy tissues, a feature that, in theory, also minimizes the risk of GVHD in NK cell-based therapies [132]. In contrast, tumor cells often evade T cell recognition by downregulating MHC I expression. This loss of “self” recognition activates NK cells' missing-self recognition pathway [133], triggering cytotoxic activity against tumors. Additionally, specific activating receptors on NK cells [134], like NKG2D, DNAM1, NKp30, and NKp46, can directly recognize ligands upregulated on tumor cells, further promoting NK cell-mediated killing. NK cells employ three primary mechanisms to eliminate tumors [135]: (i) releasing cytotoxic granules containing perforin and granzymes, (ii) secreting cytokines like IFN-γ and TNF-α to induce apoptosis or activate other immune cells, and (iii) utilizing Fc receptors to trigger antibody-dependent cell-mediated cytotoxicity. This diverse anti-tumor arsenal positions NK cells as a promising avenue for ACT.

**Figure 4.**
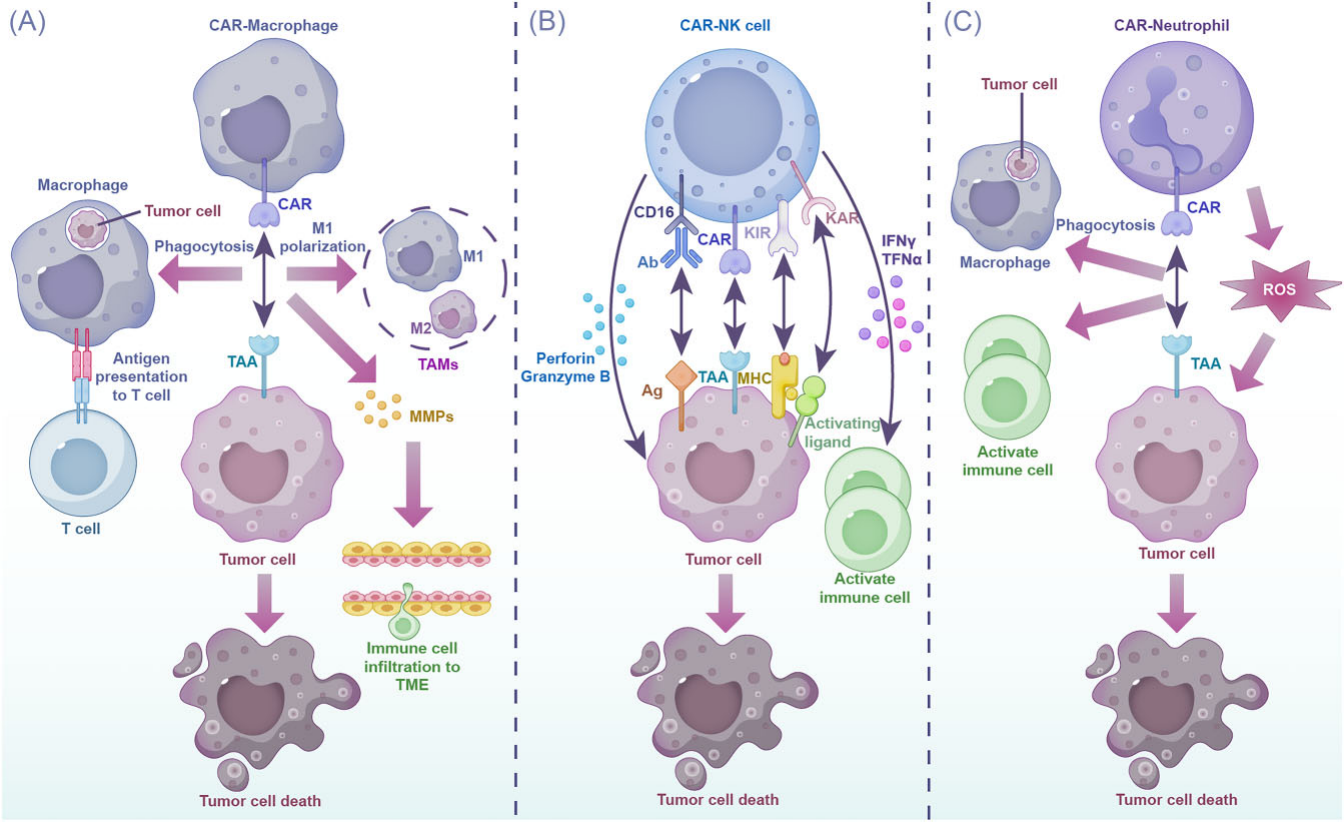
(**A**) Tumor cell eliminating mechanisms of CAR-macrophage cells. CAR-macrophage cells recognize tumor cells through the TAA. Tumor cell death is mediated by phagocytosis of tumor cells, which further present tumor antigen to T cells to induce cell*-*mediated immunity against cancer. M1 polarization and production of matrix metalloproteinases (MMPs) facilitating immune cell infiltration also induce anti-tumor activity. (**B**) Tumor cell eliminating mechanisms of CAR-NK cells. CAR-NK cells recognize tumor cells through the TAA. Tumor cell death is mediated by activation of immune cell anti-tumor cytotoxic activity through IFN-γ and TNFα secretion, and tumor cell apoptosis and necrosis activated through killer cell activatory receptors (KAR) and activating ligand recognition and perforin and granzyme B granules secretion. (**C**) Tumor cell eliminating mechanisms of CAR-neutrophil cell. CAR-neutrophils recognize tumor cells through the TAA. They kill tumor cells through reactive oxygen species (ROS)-dependent mechanisms, promoting phagocytosis and T cell-dependent anti-tumor immunity.

CAR-NK cell therapy leverages the natural cytotoxic potential of NK cells by engineering them with CARs [136]. The source of NK cells for CAR-NK therapy can vary [137], each with its own advantages and limitations. Allogeneic peripheral blood offers readily available, highly differentiated NK cells, but large-scale culturing remains challenging. Umbilical cord blood provides NK cells with high proliferation potential, but their cytotoxic activity is more limited [138]. While stem cell-derived NK cells [139] exhibit broad anti-tumor activity, concerns regarding immunogenicity and malignant transformation necessitate careful evaluation. NK cell lines like NK-92 and YT offer a readily expandable source but require pre-administration irradiation and demonstrate a short lifespan *in vivo* [140]. Selecting an appropriate CAR transfection strategy is crucial for successful CAR-NK cell production. Non-viral methods include mRNA and DNA transfection, while viral vectors encompass retroviral, lentiviral, and adeno-associated virus vectors [137]. Additionally, transposon transfection and CRISPR/Cas9 gene editing technology offer possibilities for precise genetic manipulation. Following CAR integration, CAR-NK cells are expanded *ex vivo* before patient infusion. Several factors contribute to CAR-NK cell trafficking and targeting [141]: (i) inherent chemotaxis of NK cells, (ii) cytokine signals from the TME that activate and recruit NK cells, and (iii) the specificity of the ScFv domain within the CAR, which guides recognition and binding to target tumor cells. Additionally, interactions between surface ligands on CAR-NK cells, such as CXCR3 and CX3CR1, and chemokines secreted within the TME play a role in directing NK cell migration. CAR-NK cells exert anti-tumor effects through several mechanisms [142]: (i) targeting specific tumor markers with the engineered CAR, (ii) engaging with self-antigens on tumor cells, and (iii) recognizing ligands expressed on the tumor cell surface (Fig. 4). Due to their inherently short lifespan, CAR-NK cells exhibit limited persistence *in vivo*. However, this can be mitigated by incorporating immunostimulatory cytokines [143, 144] (IL-2, IL-7, IL-21) into the treatment regimen to enhance their survival. Furthermore, repeated infusions of CAR-NK cells can be employed to maintain a sufficient effector cell population for sustained disease control.

Several surface markers are overexpressed in GC tissues, including HER2 [145], MUC1 [146], CEA [147], EpCAM [102], NKG2D ligands [148], and others. This overexpression makes them promising targets for CAR-NK cell therapy. Preclinical studies have explored the potential of CAR-NK cells for GC treatment. MSLN-CAR-NK92 cells [149], for example, specifically target MSLN-positive cancer cells *in vitro*, demonstrating enhanced anti-tumor effects and increased NK cell infiltration *in vivo*. Similarly, studies with NK-92/5.137.z cells [150], engineered with a HER2-specific second-generation chimeric antigen receptor, have shown promising results. These CAR-NK cells exhibit enhanced cytotoxicity and higher cytokine release *in vivo*, leading to the elimination of small tumors. However, the effects on larger tumors require further investigation. Current evidence suggests that combination therapy with apatinib [151], a tyrosine kinase inhibitor, can further improve NK cell infiltration and enhance tumor-killing ability. NKG2D ligand overexpression on GC cell surfaces presents another opportunity for CAR-NK therapy. NKP30-CAR-NK cells [152] have demonstrated anti-tumor efficacy against GC in preclinical studies. CDH17, a protein specifically expressed in gastrointestinal cancers, is also a potential target. Finally, non-viral CDH17 CAR-NK cells have been engineered using mRNA electroporation of Magicell-NK cells [153]. These CAR-NK cells contain a novel CDH17-specific single-chain variable fragment and a 4–1BB co-stimulatory domain. *In vitro* studies demonstrated optimal cell viability, increased expression of surface CD107a or lysosome-associated membrane protein 1 (LAMP-1) on CAR-NK cells co-cultured with CDH17-positive cancer cells, and enhanced anti-tumor responses. Taken together, these findings suggest that non-viral CDH17 CAR-NK cell therapy may be a promising approach for gastrointestinal cancers, including GC. Notably, CAR-NK therapy for GC remains in the preclinical development stage. Further research is warranted to evaluate its safety and efficacy in clinical trials.

Furthermore, the past few years have witnessed a growing interest in modified CAR-NK cells, such as BiNK and TriNK. These next-generation CAR-NK cells [154] hold promise for treating tumor heterogeneity due to their broader anti-tumor capabilities, lower toxicity profile, and reduced risk of serious adverse reactions. However, the efficacy of CAR-NK therapy in GC can be hampered by several factors within the TME [155]. These include high levels of PD-L1 expression, which can suppress NK cell activity, PD-1 expression on the NK cells themselves, and the presence of inhibitory receptors (TIM-3, LAG-3, and TIGI) on the CAR-NK cells. To overcome these limitations, combination therapy with ICIs has shown promise [156]. This approach can enhance antibody-dependent cell-mediated cytotoxicity-mediated anti-tumor activity, boost cytokine production, and inhibit NK cell apoptosis in PD-L1-positive GCs and other gastrointestinal malignancies.

### CAR-macrophages

Tumor-associated macrophages are the most abundant immune cells within the TME [157] and play a critical role in suppressing the immune response against tumors. Macrophages can be polarized into two main phenotypes: M1 and M2 [158]. M1 macrophages exhibit pro-inflammatory properties and inhibit tumor development, while M2 macrophages promote tumor growth, invasion, and metastasis [159]. In GC, the dynamic changes in the TME influence macrophage polarization. The hypoxic environment induces macrophages to secrete CXCL8, which promoted gastric erosion [160], and IL-10, which leads to M2 polarization. This creates a positive feedback loop that favors tumor progression. TAMs further suppress the anti-tumor function of CD8^+^ T cells and are associated with high expression of the immune checkpoint PD-L1 [161]. Additionally, they promote GC migration by increasing the expression of matrix metalloproteinase (MMP) genes and kindlin-2-related signaling pathways. Notably, TAM infiltration is considered an independent risk factor for advanced GC. GC tissue exhibits upregulated expression of specific factors [34] like miR210, regulator of G protein signaling 1 [162], BICCI [163], FSTL3 [164], and YKL-39 [165], which collectively promote TAM infiltration and GC development. TAMs are also closely associated with GC's resistance to chemotherapy. Given their phagocytic ability and role in immunity, macrophages offer a promising target for cancer therapy. Researchers are exploring genetically modified macrophages that can engulf tumor cells or maintain an M1 phenotype. CAR-macrophages represent a particularly exciting approach [166]. Induced pluripotent stem cell (iPSC)-derived CAR-expressing macrophage cells (CAR-iMacs) can overcome limitations of insufficient autologous macrophage numbers and disease-induced suppression [167]. Their indefinite proliferation and amenability to genetic modification make them a valuable source for immunotherapy. CAR-iMACs [168] have shown promising results in pre-clinical models, demonstrating safety and the ability to engulf cancer cells. While other sources like monocytes from peripheral blood mononuclear cells or bone marrow macrophages offer easier accessibility [169], they are limited in terms of expansion and genetic modification. The structure of CAR-M follows a similar design to traditional CAR T cells [170], with an extracellular antigen-binding domain, a hinge region, a transmembrane domain, and an intracellular signaling domain. The key difference lies in the selection of cytoplasmic domains that promote phagocytosis [166, 171], such as Tlr4, Megf10, Fc receptor, and common γ subunits (FcRɣ) of MerTK (CD3ζ, CD28, 4–1BB, Megf10, MerTK, PI3K, CD86, CD147, TLR2, TLR4, and TLR6). Building upon the initial success of CAR-iMACs, researchers have developed second-generation CAR-M designs with enhanced functionality. The CD3ζ-TIR-CAR-iMAC [171] incorporates a dual-signal CAR structure utilizing both CD3ζ and toll/interleukin-1 receptor (TIR) domains in iPSC-derived macrophages. This design demonstrates superior anti-tumor activity and a stronger M1 polarization phenotype compared to single-signal CD3ζ CAR-M. Gene transfer for CAR-M therapy presents a unique challenge due to the macrophages's natural ability to clear viruses. Traditional viral transfection methods have low efficiency. Currently, feasible options include adenovirus vectors with modified serotypes [172] (such as Ad5/F35) and lentiviral transfection using HIV-2 derived Vp proteins [173] to degrade SAMHD1, a restriction factor. Non-viral transfection methods are also being explored and generally align with those used for CAR-T cell therapy [174]. Within the TME, CAR-M recognizes target antigens on cancer cells, triggering a multifaceted response [175]. First, CAR-M engulfs and eliminates the targeted tumor cells. Additionally, CAR-M activates surrounding antigen-presenting cells, which then present tumor antigens to T cells, stimulating a broader anti-tumor immune response. Furthermore, CAR-M functions to anchor macrophages within the tumor, promoting their M1 polarization and reprogramming the TME. This includes the production of MMPs to degrade the tumor's extracellular matrix, facilitating immune cell infiltration. CAR-M also releases cytokines that shape the TME and promote further M1 polarization of macrophages within the tumor (Fig. 4). Compared to CAR-T and CAR-NK therapies, CAR-M offers several advantages in the context of solid tumors. These include sufficient cell infiltration, robust phagocytic ability, and the capacity to reshape the tumor matrix [176]. CAR macrophages designed with the CAR-147 intracellular domain activate MMPs within tumor models, facilitating T cell infiltration by degrading the basement membrane, a natural barrier [177]. This design also enables CAR-dependent MMP secretion within tumors, potentially synergizing with other therapies like CAR-T cell therapy for enhanced anti-tumor effects. Preclinical studies support the efficacy of CAR-M. Zhang *et al*. [167] demonstrated the *in vitro* phagocytosis of CD19-expressing leukemia cells and mesothelin-expressing ovarian/pancreatic cancer cells by CAR (CD19)-iMac and CAR (meso)-iMac cells, respectively. These CAR-M designs also exhibited anti-tumor activity in *in vivo* models. Similarly, HER2/CD47 CAR-M has been validated for its phagocytic ability and multifaceted anti-tumor effects on ovarian cancer cells. In the context of GC, Dong *et al*. [178] developed a promising HER2-FcεR1γ-CAR (HF-CAR) targeting HER2-positive GC cells. These CAR-M cells derived from human gastric peritoneal macrophages demonstrated excellent anti-tumor efficacy and safety in pre-clinical studies. Furthermore, research suggests that CAR-M therapy can improve the TME, leading to enhanced responses to chemotherapy drugs. For instance, oxaliplatin can promote M1 polarization, and combination therapy with CAR-M can enhance anti-tumor efficacy and prolong survival [178]. The complexity and cost of *in vitro* CAR-M manufacturing have spurred research into *in vivo* programming strategies. Kang *et al*. [179] demonstrated effective tumor suppression by delivering CAR and IFN-γ gene-containing nanoparticles loaded with macrophages directly into the body, inducing CAR-M1 macrophages *in situ*. Beyond CAR-M, other macrophage-based immunotherapies hold promise [180]. These include adoptive cell therapy using iPSC-derived macrophages, nanoparticle-loaded macrophages, *ex vivo* polarized and/or adoptively transferred macrophages, and surface anchoring engineering of macrophages.

### CAR-neutrophils

Similar to TAMs, tumor-associated neutrophils (TANs) exhibit phenotypic plasticity and can be categorized into two main subsets: anti-tumor N1 and pro-tumor N2 [181]. The phenotype of N1 TANs is similar to that of M1 macrophages, as they are associated with IFN-γ and granulocyte-macrophage colony-stimulating factor (GM-CSF), and they demonstrate a direct anti-tumor activity through reactive oxygen species (ROS)-dependent mechanisms [182]. Such neutrophils can also activate other immune cells, including T lymphocytes, to promote T cell-dependent anti-tumor immunity and the polarization of unconventional T cells towards the αβ subtype [183]. Conversely, N2 TANs exhibit an M2-like phenotype, characterized by high expression of ARG1, CCL17, and CXCL14 [184, 185]. These cells promote tumor development through various mechanisms, including VEGF production, extracellular matrix remodeling, and the release of ROS and reactive nitrogen species (Fig. 4). Additionally, N2 TANs mediate immune suppression. The success of CAR-M in maintaining M1 polarization and enhancing anti-tumor responses suggests a potential role for engineered neutrophils (CAR-N) in cancer therapy [186]. However, the short lifespan and resistance of neutrophils to gene editing pose challenges for CAR-N development. Despite these limitations, pre-clinical studies have shown promise. Chang *et al*. [187] designed CLTX-T-CAR neutrophils derived from human pluripotent stem cells. These cells exhibit typical neutrophil phenotypes and enhanced anti-tumor cytotoxicity via phagocytosis, ROS release, and neutrophil extracellular trap formation. Additionally, CLTX-T-CAR neutrophils can naturally penetrate the blood–brain barrier and target MMR2, demonstrating anti-glioblastoma multiforme effects. Notably, unlike CAR-NK cells, CLTX-T-CAR neutrophils retain their anti-tumor phenotype, high trafficking capacity, and cytotoxicity even within a simulated hypoxic tumor microenvironment. Harris *et al*. [188] demonstrated the potent cytotoxic effects of CAR-N targeting the prostate-specific membrane antigen expressed on LNCaP cells, a human prostate cancer model. These CAR-N cells were derived from human pluripotent stem cells (hPSCs) modified via CRISPR-Cas9 gene editing. Furthermore, Chang *et al*. [189] employed CAR-N to deliver and release the tumor microenvironment-responsive nanodrug Tirapazamine (TPZ), significantly inhibiting tumor growth and prolonging animal survival in glioblastoma xenograft models. This combination of chemoimmunotherapy using a biomimetic CAR-neutrophil drug delivery system highlights the potential for enhanced anti-tumor activity and safety. While pre-clinical studies for GC are not yet available, CAR-N therapy presents a promising new avenue for GC treatment, particularly when combined with GC immunosurveillance and immunomodulation strategies targeting the HER2 surface marker.

## Adverse events of immunotherapy

Immune therapy has revolutionized cancer treatment, offering renewed hope and treatment options for patients with advanced or treatment-refractory cancers. This approach harnesses the power of the patient's own immune system to combat tumors. However, a growing concern with the use of immune therapy is the emergence of immune-related adverse events (irAEs). These toxicities differ significantly from those associated with traditional chemotherapy. irAEs are thought to be linked to autoimmune reactions, cytokine-mediated inflammation, and patient-specific risk factors. The occurrence of irAEs can significantly compromise a patient's quality of life and, in severe cases, even lead to death. Paradoxically, some studies suggest a positive correlation between the occurrence of irAEs and overall patient outcomes. This underscores the critical need to understand the complex relationship between irAEs and treatment efficacy. Additionally, enhancing awareness of the clinical presentation, early diagnosis, and effective management of these immune-related toxicities is crucial for optimizing patient care. The rest of this review will focus on irAEs associated with ICI therapy and ACT.

### Adverse events of ICI

ICI therapy potentiates the anti-tumor function of T cells by blocking inhibitory pathways within the immune system. While this approach leads to sustained anti-tumor responses in cancer patients, it can also result in overactivation of the immune system. This overactivation can lead to attacks on healthy tissues and excessive immune responses, irAEs can affect nearly any organ but they primarily affect the skin, liver, gastrointestinal tract, and endocrine organs. The National Cancer Institute's Common Terminology Criteria for Adverse Events (CTCAE) provides a standardized framework for assessing the severity of irAEs, grading them, and guiding treatment decisions. Grade 3 AEs are considered moderate to severe, requiring hospitalization but not life-threatening. Grade 4 AEs are life-threatening, and grade 5 indicates death. The severity of irAEs is influenced by several factors [190, 191], including the specific drug used, dosage, patient's immune response profile, and tumor type. In the context of GC, ICI therapy has a reported incidence of all-grade treatment-related adverse events (TRAEs) of 54.5% [192], with fatigue, alanine aminotransferase increase, hepatitis, and pneumonitis being common grade 3 or higher TRAEs. A retrospective analysis of 402 cases treated with PD-1 inhibitors for advanced GC revealed that skin toxicity, endocrine toxicity, and gastrointestinal toxicity were the most common adverse effects, occurring at lower toxicity levels. Hepatic and pulmonary toxicities were less frequent but tended to be associated with higher toxicity levels. More than 50% of patients treated with tislelizumab, toripalimab, or sintilimab experienced irAEs [193]. In a phase I study of avelumab monotherapy in GC patients, 15.3% experienced irAEs, with only 2% encountering grade 3 irAEs and no irAE-related deaths reported [194]. A phase II clinical trial testing trastuzumab and nivolumab in combination with either ipilimumab or mFOLFOX6 for previously untreated HER2-positive esophagogastric adenocarcinoma found a higher incidence of irAEs and poorer treatment outcomes with ipilimumab combination therapy compared to mFOLFOX6 [195]. Furthermore, a model-based analysis by Shulgin *et al*. [196] on irAE rates of CTLA-4 and PD-1 inhibitors in cancer patients demonstrated dose/exposure dependence, with PD-1/PD-L1 inhibitors showing no such dependence. In contrast, CTLA-4 inhibitors exhibited a strong dose/exposure dependence, with higher concentrations correlating with increased rates of grade 3 and 4 adverse events, consistent with current clinical research [197]. However, no specific dose-ranging study for GC was identified. Compared to chemotherapy alone, ICI therapy can potentially reduce adverse events [192]. However, combination therapy with ICIs often increases the risk of any-grade AEs [198], grade 3 or higher AEs, and treatment discontinuation due to TRAEs such as alanine aminotransferase elevation, palmoplantar erythrodysesthesia syndrome, nausea, and leukopenia. Park *et al*. proposed that the combination of nivolumab and ipilimumab can improve overall survival in third-line GC treatment, but at the cost of higher toxicity [199]. Conversely, the research by Huang *et al*. [200] demonstrated that the combination of apatinib (a VEGFR-2 inhibitor) and nivolumab offered a significant improvement in overall survival with fewer TRAEs, potentially representing the optimal treatment approach for third-line GC [199]. Therefore, selecting the most appropriate ICI drug, dosage, and combination regimen is crucial to balance treatment efficacy with the minimization of irAEs. Current management strategies for irAEs focus on early identification, prompt intervention, and minimizing the risk of fatal complications. Understanding risk factors and identifying potential predictive molecules for irAEs is an essential area of ongoing research. A study by Chennamadhavuni *et al*. identified several factors that influence the risk of irAEs in patients receiving ICI therapy [201]. These factors include patient age, sex, medication history, and the presence of pre-existing organ-specific diseases. Clinicians should carefully consider these factors before initiating ICI therapy [201]. Furthermore, researchers are exploring the use of biomarkers to predict the occurrence and severity of irAEs. Several potential biomarkers have been identified. Female sex, antibiotic use, a high neutrophil-to-lymphocyte ratio following treatment, and a higher baseline level of circulating tumor cells are associated with an increased risk of irAEs. Conversely, a lower baseline prognostic nutritional index, a body mass index ≥ 25 kg/m^2^, and higher post-treatment lactate dehydrogenase levels may indicate a higher risk of developing more severe irAEs [202].

### Adverse events of ACT

Cellular therapy for tumors in clinical trials has shown promise, but it can also lead to various adverse reactions affecting the entire body. One of the most common immune system toxicities associated with CAR-T therapy is CRS. CRS is characterized by a series of clinical symptoms, including nausea, headache, rapid heart rate, low blood pressure, rash, and difficulty breathing, all resulting from the release of cytokines by the infused T cells. The American Society for Blood and Marrow Transplantation (ASTCT) grades CRS severity on a five-point scale [203], with grade 3 being the cutoff for requiring specific interventions like anti-cytokine therapy. While grades 1 and 2 are considered low-risk, grade 3 or higher CRS necessitates treatment, and grade 5 indicates death. A meta-analysis revealed that 78% of patients with diffuse large B-cell lymphoma (DLBCL) treated with CAR-T therapy experienced CRS, with 6% developing severe CRS. The structures of the CAR-T cells themselves seem to influence the risk of CRS and neurotoxicity. Products incorporating the 4–1BB costimulatory domain have demonstrated a higher safety profile [204] compared to those using the CD28 domain. A case report documented fatal events in metastatic melanoma patients treated with melanoma antigen recognized by T cells 1 (MART-1) TCR-T cell therapy, where cytokine release syndrome induced by activated T cells was believed to be the primary cause of death [205]. Additionally, the risk of CRS appears to be linked to the specific CAR-T cell construct, the initial quantity of infused T cells, and their subsequent proliferation *in vivo*. Patients treated with 4–1BB co-stimulated CAR-T cells typically experience CRS later than those receiving CD28 co-stimulated CAR-T cells [206]. Early elevation of cytokines and a high tumor burden are considered high-risk factors for severe CRS development. The excessive release of cytokines by activated T cells is responsible for CRS. On the other hand, these ICI treatment options are sometimes so effective that the rapid destruction of tumor cells may often trigger tumor lysis syndrome. Essentially a metabolic disorder, tumor lysis syndrome can lead to imbalances in electrolytes like potassium, phosphate, and uric acid, and even acute kidney injury. While more frequent in immunotherapy for blood cancers, it can also occur in solid tumors [207].

Immune effector cell-associated neurotoxicity syndrome (ICAN) is the second most common serious reaction to CAR-T therapy. Patients with ICAN often experience encephalopathy, aphasia, delirium, tremors, and seizures. These symptoms typically resolve within 3–4 weeks after treatment, but severe ICANs can lead to fatal diffuse cerebral edema [208]. The CARTOX-10 screening tool is used to grade ICAN severity. Grades 1 and 2 are considered mild to moderate, with no increased intracranial pressure, seizures, or motor weakness. Grade 3 or higher ICANs are considered severe, with increased intracranial pressure, and partial or generalized seizures, and can be fatal (grade 5). A study examining 19–28z CAR T-cell therapy for relapsed or refractory acute lymphoblastic leukemia found that 62.3% of patients experienced some level of neurotoxicity. Similar to CRS, high initial tumor burden, early cytokine elevation, and high CAR-T cell expansion were associated with a greater risk of severe ICANs. Mechanisms underlying fatal ICANs are thought to involve bone marrow-driven systemic inflammation leading to endothelial activation and blood–brain barrier dysfunction [209]. Unlike CRS, CD28 co-stimulated CAR-T cells seem to present a higher risk for ICAN development [209]. Current evidence suggests no neurotoxicity or CRS associated with CAR-NK therapy compared to CAR-T. Furthermore, phase I trials of claudin18.2 CAR-T therapy for gastrointestinal cancers reported that 100% of grade 3 or higher adverse events were hematological toxicities, with leukopenia affecting 83.8% of patients [82].

Beyond on-target tumor toxicity, off-target effects can have serious consequences. A case in point is the fatal cardiac toxicity observed in patients receiving autologous infusion of enhanced affinity T-cell receptor targeting the testicular antigen MAGE-A3. TCR-T therapy has also been implicated in off-target toxicities leading to cardiac issues [210] and acute respiratory distress syndrome [211]. The rates and severity of these toxicities vary across different multicenter trials. This variability may be associated with factors such as trial design, medication use, individual patient differences, underlying disease types, toxicity grading systems, and the specific type of CAR-T cell construct used.

## Nanotechnology

Recent years have witnessed a growing interest in integrating nanotechnology with immunotherapy, leading to significant breakthroughs. Nanoparticles, acting as customizable carriers, offer precise modulation of immunotherapy through controlled particle shape, size [212], and surface charge [213]. Biodegradable polymer nanoparticles like poly(lactic-co-glycolic acid), PLGA [214], along with metal and inorganic nanoparticles, have found extensive use in immunotherapy. These nanoparticles can modify CAR-T cells *in vivo* [215], regulate CD4^+^ T cell function [216], inhibit the expression of tumor-associated immunosuppressive cells [217], and control the release of immunosuppressive agents like IL-2 and transforming growth factor-β (TGF-β) [218]. This ultimately normalizes the immune microenvironment and enhances immune infiltration. Furthermore, nanoparticles serve a unique role as immunopotentiators [219] and drug carriers [220]. They can deliver antigens and immune adjuvants, facilitating antigen presentation and ultimately activating the immune system. Nanoparticles enhance antigen uptake and processing efficiency by leveraging their distinct properties, including surface functionalization and targeting effects. For instance, PLGA nanoparticles, due to their size similarity to microbial pathogens [219], can promote antigen presentation by mimicking the way immune cells engulf these pathogens. Additionally, nanoparticle-based platforms like synthetic high-density lipoprotein nanodiscs demonstrate remarkable efficacy. These nanodiscs deliver neoantigens and Toll-like receptor 9 agonists to antigen-presenting cells in draining lymph nodes, resulting in a significant increase in neoantigen-specific cytotoxic CD8^+^ T lymphocytes [221]. When combined with ICIs, nanodisc vaccines in colon cancer and B16F10 melanoma mice exhibited superior tumor regression rates [222] compared to single-dose nanodisc vaccines. This highlights the potential of nano-vaccines, which exhibit comparable efficacy, safety, and anti-tumor response to refractory cancer immunotherapy compared to advanced cancer vaccines. Moreover, researchers are designing nanoparticles to achieve combined photothermal therapy and ICI therapy. These nanoparticles assemble photothermal agents and immune activators, resulting in superior and sustained tumor response alongside long-term immune memory effects [223].

Nanoparticles have demonstrably enhanced immunotherapy outcomes, specifically in the context of GC. Nanotechnology-based cancer vaccines offer exciting new prospects for GC immunotherapy. For instance, the MAGE-A4 protein and cholesterol-bearing hydrophobized pullulan nanoparticle complex vaccine has induced MAGE-A4-specific humoral immune responses in advanced cancer patients, including those with GC (alongside esophageal and lung cancers) [224]. Similarly, nanovaccines comprising epitope peptides from CD4^+^ and CD8^+^ T cells have effectively stimulated anti-tumor immune responses in GC patients. Furthermore, the properties of nanomaterials can directly impact the survival of gastrointestinal cells [225, 226]. Wu *et al*. developed nanometer-sized poly(phenylene ethynylene)-based antimicrobial agent [227], which activates both external and internal apoptotic pathways in various cancers, including GC, by leveraging cancer cell amino acid metabolic dependence and the ROS generation ability of its multi-nanopore cores. Nanoplatforms loaded with cisplatin have also shown promise in GC treatment [228]. These platforms increase cisplatin accumulation in GC tissues, enhance its overall toxicity, and potentially overcome drug resistance. Li *et al*. [229] demonstrated a synergistic effect by combining nanosystems with both chemotherapy and immunotherapy. Their nanodrug delivery system, DOX@aiPS-DCexo, enhanced chemotherapy efficacy while simultaneously releasing a significant number of tumor antigens, thus stimulating the immune system. Beyond treatment, nanoparticles can be combined with imaging technology for immune detection and evaluation. This allows for real-time monitoring of therapeutic drug release and distribution, evaluation of therapeutic efficacy, and guidance for treatment plan adjustments. This approach enables a more accurate assessment of immunotherapy response and prognosis, ultimately providing a basis for personalized treatment in GC.

## Conclusion

Immunotherapy has made significant strides in the clinical management of GC, with ICIs becoming established and reliable first-line treatment options for patients with advanced GC. Combination therapy, offering superior efficacy and reduced toxicity, has become the standard approach. This personalized approach relies on biomarkers such as MSI, PD-L1 combined positive score, EBV status, tumor mutational burden, circulating tumor DNA, CTCs, and *H. pylori* status. These biomarkers can predict patient response, guide treatment planning, and assess therapeutic efficacy in ICI treatment. Therefore, future research should focus on comprehensively considering these biomarkers alongside individual patient differences. This includes selection of optimal ICI drugs, dose determination, and effective drug pairing strategies. This focus will ultimately achieve optimal therapeutic effects while minimizing immune-related adverse events.

ACT presents another promising avenue for GC immunotherapy. Advancements in cell therapy technologies, including CAR-T, TILs, TCR-T, CAR-M, and CAR-NK cells, offer new therapeutic possibilities for GC patients. However, applying ACT in GC faces challenges stemming from the TME. These challenges include a lack of well-defined tumor-specific antigens, tumor antigen escape mechanisms, suppression of immune cell infiltration, immunosuppressive enzymes, stromal fibroblasts, the extracellular matrix, tumor angiogenesis, and hypoxia. Furthermore, issues associated with the therapeutic cells themselves, such as targeted and off-target toxicities observed with CAR-T and CAR-M therapies, can hinder treatment efficacy. Researchers are exploring various strategies to address these challenges and enhance ACT efficacy. One approach is the development of dual CAR strategies to overcome tumor antigen heterogeneity, antigen loss variants, and limitations of CAR-T cells in antigen-negative tumors, while simultaneously avoiding attacks on healthy tissues. CAR-NK cell therapy is considered a promising alternative to CAR-T therapy due to its potential for effective tumor elimination with lower toxicity. This approach leverages both CAR-dependent and NK cell receptor-dependent mechanisms. However, further research is warranted to investigate the long-term *in vivo* anti-tumor effects of CAR-NK therapy in GC.

Future research endeavors should continuously optimize existing treatment modalities, explore optimal combination regimens, and develop novel immunotherapy approaches. These approaches include gene editing therapies based on CRISPR technology and expanded nanotechnology applications. Looking ahead, research and development efforts for new immunotherapies like CAR-NK, CAR-M, and CAR-N cells are still in their early stages. We anticipate further preclinical studies and multi-omics clinical trials to unlock breakthroughs in GC treatment.
